# *Cryptococcus neoformans* Hyperfilamentous Strain Is Hypervirulent in a Murine Model of Cryptococcal Meningoencephalitis

**DOI:** 10.1371/journal.pone.0104432

**Published:** 2014-08-05

**Authors:** Marianna Feretzaki, Sarah E. Hardison, Floyd L. Wormley, Joseph Heitman

**Affiliations:** 1 Department of Molecular Genetics and Microbiology, Duke University Medical Center, Durham, North Carolina, United States of America; 2 Department of Biology, The University of Texas at San Antonio, San Antonio, Texas, United States of America; V.P.Chest Institute, India

## Abstract

*Cryptococcus neoformans* is a human fungal pathogen that causes lethal infections of the lung and central nervous system in immunocompromised individuals. *C. neoformans* has a defined bipolar sexual life cycle with **a** and α mating types. During the sexual cycle, which can occur between cells of opposite mating types (bisexual reproduction) or cells of one mating type (unisexual reproduction), a dimorphic transition from yeast to hyphal growth occurs. Hyphal development and meiosis generate abundant spores that, following inhalation, penetrate deep into the lung to enter the alveoli, germinate, and establish a pulmonary infection growing as budding yeast cells. Unisexual reproduction has been directly observed only in the *Cryptococcus* var. *neoformans* (serotype D) lineage under laboratory conditions. However, hyphal development has been previously associated with reduced virulence and the serotype D lineage exhibits limited pathogenicity in the murine model. In this study we show that the serotype D hyperfilamentous strain XL280α is hypervirulent in an animal model. It can grow inside the lung of the host, establish a pulmonary infection, and then disseminate to the brain to cause cryptococcal meningoencephalitis. Surprisingly, this hyperfilamentous strain triggers an immune response polarized towards Th2-type immunity, which is usually observed in the highly virulent sibling species *C. gattii*, responsible for the Pacific Northwest outbreak. These studies provide a technological advance that will facilitate analysis of virulence genes and attributes in *C. neoformans* var. *neoformans*, and reveal the virulence potential of serotype D as broader and more dynamic than previously appreciated.

## Introduction

*Cryptococcus neoformans* is an opportunistic fungal pathogen that is distributed worldwide. Pathogenic *Cryptococcus* lineages comprise two main species: *Cryptococcus neoformans* and *Cryptococcus gattii*. *C. neoformans* is classified into two varieties: var. *grubii* (serotype A) and var. *neoformans* (serotype D). The serotype A variety is the most common cause of infection, typically of the central nervous system in immunocompromised individuals, including organ-transplant patients and HIV-infected individuals. Cryptococcal meningitis is an AIDS-defining illness in 30% of HIV/AIDS presentations and it is a major cause of mortality in AIDS patients in HIV/AIDS endemic regions, such as Southeast Asia and Sub-Saharan Africa [1], [2]. Serotype D, which is characterized by lower pathogenicity, diverged from serotype A ∼18.5 million years ago and is more prevalent in Europe [3]. On the other hand, *C. gattii* infects healthy individuals and is responsible for the ongoing *Cryptococcus* outbreak on Vancouver Island and in the Pacific Northwest in Canada and the United States [4], [5], [6].

*C. neoformans* has a defined life cycle and grows in the environment as a haploid budding yeast with α (common) and **a** (rare) mating type cells. Under nutrient limiting conditions or in response to V8/inositol it has the ability to mate and undergo a dimorphic transition from vegetative yeast to hyphal growth [7]. Hyphal development can occur between cells of the opposite mating type (bisexual reproduction) or cells of one mating type (unisexual reproduction), resulting in production of basidia, where meiosis occurs to generate haploid spores [8]. Unisexual reproduction has been directly observed under laboratory conditions for serotype D isolates. Population genetic studies support the hypothesis that unisexual reproduction also occurs in nature in *Cryptococcus neoformans* var. *grubii* and *C. gattii*, and may be responsible for the evolution of highly pathogenic strains [9], [10], [11], [12], [13], [14], [15], [16].

Cryptococcal infections are thought to be acquired by inhalation of infectious propagules from the environment. Spores are of an ideal size to penetrate and colonize the alveoli of the lung and are proven to be pathogenic [17], [18], [19]. *C. neoformans* can colonize the host's respiratory tract for years and when host immunity is compromised the fungal cells disseminate hematogenously, cross the blood brain barrier, and cause cryptococcal meningoencephalitis. The morphology of the fungus inside the host varies from small round yeast cells to titan cells, which are thought to play roles in survival and evasion of the immune system during infection [20], [21]. However, hyphae or pseudohyphae are rare, as hyphal growth is suppressed by the physiological conditions of the host (high temperature, moisture, and high CO_2_ levels) [22], [23], [24], [25], [26], [27].

The mechanism via which infections are acquired is universal for all *Cryptococcus* species; however, the immune response of the host can differ significantly between var. *neoformans*, var. *grubii*, and *C. gattii*. In immunocompetent individuals the serotype D spores or yeast propagules that reach the lung are cleared by a protective immune response characterized by elevated expression of Th1-type cytokines (IFN-γ, TNF-α, IL-12, IL-2) and high concentrations of macrophages and dendritic cells that successfully clear the infection in the murine model [28], [29], [30], [31]. In contrast, *C. gattii* infections in mice trigger a non-protective immune response through high levels of Th2-associated cytokines (IL-4, IL-5, IL-9, IL-10, and IL-13) [30], [32], [33], [34]. Dong *et al*. hypothesized that *C. gattii* infects healthy individuals because high levels of IL-4 inhibit neutrophil migration to the lung (which is crucial for protective immunity), allowing yeast cells to survive longer inside the host and disseminate successfully to the brain [34]. Interestingly, experimental pulmonary infections with the serotype A strain H99α elicit different types of immunity in different mouse models. In BALB/c and CBA/J mice H99α triggers a Th-2 type immunity that is related to changes in lung function and results in a fatal outcome [31], [35], [36], while in the C57BL/6 mouse model serotype A induces a mixed immune response that alternates between Th1 and Th2 during the course of the infection that greatly affects the outcome of the disease [32], [37]. Thus, for *Cryptococcus* cells to survive inside the host it may be crucial to induce a less protective immune response.

Population genetics data have shown that unisexual reproduction may occur in nature for serotype A and *C. gattii* species, although the unisexual cycle has not been directly observed under laboratory settings. Thus, although unisexual reproduction may play a role in virulence, through production and dispersal of infectious spores, it has only been observed in the lower pathogenicity group of serotype D. Moreover, many serotype D strains exhibit limited filamentation and produce few spores. Previous studies identified a unique hyperfilamentous serotype D strain that can generate abundant hyphae and spores via unisexual reproduction [38]. It was also recently reported that this strain (XL280α) and a congenic partner of opposite mating type (XL280**a**) cause significant mortality in a murine model [39], [40] compared to previous animal studies on serotype D [41], [42].

In light of these findings, we studied the virulence of the hyperfilamentous strain XL280α in a murine model compared with its well-established parent JEC21α. Although the two serotype D strains are phenotypically similar, we found that XL280α is hypervirulent compared to JEC21α. We also found that XL280α causes both pulmonary and central nervous system infections. Although both of these strains grow as budding yeasts inside the host, XL280 cells were significantly larger and oblong, with an oval like shape. Surprisingly, our study revealed high levels of Th2-type cytokines in the lungs of XL280α infected mice, indicative of a less protective immune response, compared to mice infected with JEC21α. These findings offer new insights into the virulence and pathogenesis of *C. neoformans* strains.

## Materials and Methods

### Ethics statement

All of the animal experiments were performed in the Division of Laboratory Animal Resources (DLAR) facilities at Duke University Medical Center (DUMC). All of the mouse experiments were performed according to the guidelines of NIH and the Duke University Institutional Animal Care and Use Committee (IACUC). The animal experiments were reviewed and approved by the DUMC IACUC under protocol number A266-08-10.

### Strains and media

The strains used in this study were *Cryptococcus neoformans* serotypes A and D. For phenotypic analysis we used the sequenced serotype A laboratory strain H99α and a mutant, *cap59*Δ, defective in capsule production [43]. XL280α is a serotype D strain and is a sequenced progeny of the two sibling strains B3501α and B3502**a**, and related to the congenic pair JEC21α/JEC20**a** genome reference sequence strains [38], [39], [40], [44], [45]. The *ste7*Δ and *cpk1*Δ pheromone signaling deletion mutants were derived in the XL280α background [46], [47]. The XL280α *crg1* mutant has a T-DNA insertion in the coding region that compromises expression of the gene [47]. All strains were maintained on yeast extract-peptone-dextrose (YPD) media. Mating cultures were performed on 5% V8 juice agar medium (pH = 5 or 7).

### Phenotypic analysis

To examine the virulence phenotypes of the strains *in vitro*, cells were grown overnight in liquid YPD cultures at 30°C. The next day the cells were washed with sterile water and adjusted to an equivalent cell density. 10-fold serial dilutions were spotted on YPD at 30°C and 37°C, on YPD +1 µg/ml FK506 at 30°C and 37°C, and on YPD +8 µg/ml fluconazole (FLU) at 30°C. To examine melanin production the cells were spotted on niger seed and L-DOPA plates and incubated for 7 days. To visualize the capsules, equal numbers of the cells were diluted into capsule inducing media (Dulbecco's modified Eagle's media with 25 mM NaHCO_3_), incubated for 7 days at 30°C, and screened by microscopy using India Ink. To estimate shed GXM, cells were grown to saturation in capsule inducing media for 7 days and then incubated at 70°C for 15 min (to heat kill the cells, release capsule, and denature protein enzymes), centrifuged at 1,600 g for 5 minutes, and the supernatant containing shed capsule was filtered with a 0.2 µm filter. Equal volumes from each strain were run on a 0.6% agarose gel at 25 volts for 12 hours and transferred onto a nylon membrane using Southern blot techniques [48]. The membrane was blocked with 5% milk in TBST (1× Tris-buffered saline plus 0.1% Tween) and probed with the monoclonal antibody (MAb)18b7 (1∶1,000 dilution) overnight at 4°C [49]. The membrane was washed with TBST, incubated with a secondary anti-mouse peroxidase-conjugated antibody (1∶10,000 dilution), washed again with TBST, and developed with ECL Prime western blotting detection reagent (Amersham).

### Murine infection and tissue dissection

*Cryptococcus* strains were grown overnight in liquid culture in YPD medium. The next day the cells were washed twice with sterile PBS, counted with a hemocytometer, and diluted to an appropriate cell density (1×10^6^ cells/ml). To prepare the yeast/hyphae inocula, the cells were spotted on V8 media and incubated for 8 days in the dark at room temperature. The colonies were scraped off the plates, vortexed, washed with PBS, counted with a hemocytometer (yeast cells and spores), and adjusted to a density of 1×10^6^ cells/ml. The intranasal instillation method was performed as previously described [50]. Briefly, groups of 10 female DBA mice (6- to 8-weeks old) were anesthetized by intraperitoneal injection of Phenobarbital (Nembutal) (37 mg/kg), and infected intranasally with 1×10^6^ cells/ml in 50 µl PBS. The cell density of the inocula was confirmed by plating serial dilutions and counting CFUs following infection. The animals were monitored twice daily through the course of the experiments. The animals showing signs of morbidity (weight loss, extension of the cerebral portion of the cranium, imbalance, gait changes, paralysis, seizures, convulsions, or coma) were sacrificed by CO_2_ inhalation. To determine the fungal burden in the infected tissues, the lungs and brains of the euthanized animals were removed, weighed, transferred to 15 ml Falcon tubes containing 2 ml of PBS, and homogenized for 10 sec at 13,600 rpm/min (Power Gen 500, Fisher Scientific). The homogenates were serially diluted and multiple dilutions were plated onto YPD media containing 100 µg/ml chloramphenicol. The plates were incubated at 30°C for 48 to 72 hrs to enumerate CFUs per gram of lung and brain.

### Cytokine analysis

Mice were infected with strain XL280 or JEC21 via intranasal instillation. At 3, 7, and 10 days post infection lung tissues were excised, homogenized in 1 ml PBS, and 50 µl were diluted and plated on YPD to determine fungal burden at designated time points, as described previously. 1 ml of 2× protease inhibitor buffer (containing PBS, Complete Protease tablets (Roche) and 0.05% Triton X100) was added and the homogenate was centrifuged at 3,500 RPM for 15 min at 4°C. Cytokine production in the pulmonary tissues was measured using the Bio-Plex Protein Array System (Luminex-based technology) (Bio-Rad Laboratories, Hercules, CA). The supernatants of the homogenized lungs were assayed for the presence of interferon (IFN)-γ, interleukin (IL)-1α, IL-1β, IL-2, IL-4, IL-5, IL-10, IL-12 p70, IL-17, tumor necrosis factor (TNF)-α, and granulocyte-colony stimulating factor [G-CSF] levels as well as chemokines (macrophage inflammatory protein [MIP]-1α (CCL3), MIP-1β (CCL4), macrophage chemoattractant protein [MCP]-1 (CCL2), and keratinocyte-derived chemokine (KC) (CXCL1)).

### Histopathology

Lungs of infected DBA mice were inflated and harvested in 10% neutral buffered formalin at 3, 7, and 10 days post infection. The fixed tissues were embedded in paraffin, cut into 5 µm sections and stained with hematoxylin and eosin (H&E), mucicarmine, or Periodic acid–Schiff (PAS stain) at the Research Histology Laboratory, Department of Pathology, Duke University Medical Center. The slides were visualized by light microscopy and the images were processed using MetaMorph Premier at the Duke University light microscopy core facility.

### Statistics

For the virulence assays, the survival rates were plotted against time using Kaplan-Meier survival curves. The P values were calculated with the Log-rank (Mantel-Cox) test and a *p* value of <0.05 was considered significant. Cytokine and chemokine data were derived from four independent experiments and the *p* values were calculated using two-way ANOVA statistical analysis. A *p* value of <0.05 was considered significant. All statistical analysis was done using Prism 4.0 (GraphPad software, La Jolla, CA, USA).

## Results

### Phenotypic characterization of XL280

To investigate the impact of hyphal development during unisexual reproduction we utilized the serotype D hyperfilamentous strain XL280α. XL280α is a haploid progeny of two well-validated and sequenced sibling strains, a hypo-filamentous isolate B3501α and a self-filamentous isolate of strain B3502**a** (which is congenic with the congenic pair JEC21α/JEC20**a**) [38], [44], [51]. In previous studies we sequenced the XL280α genome and found that it shares 99.88% overall genetic identity with the JEC21α parental strains.

**Figure 1:**
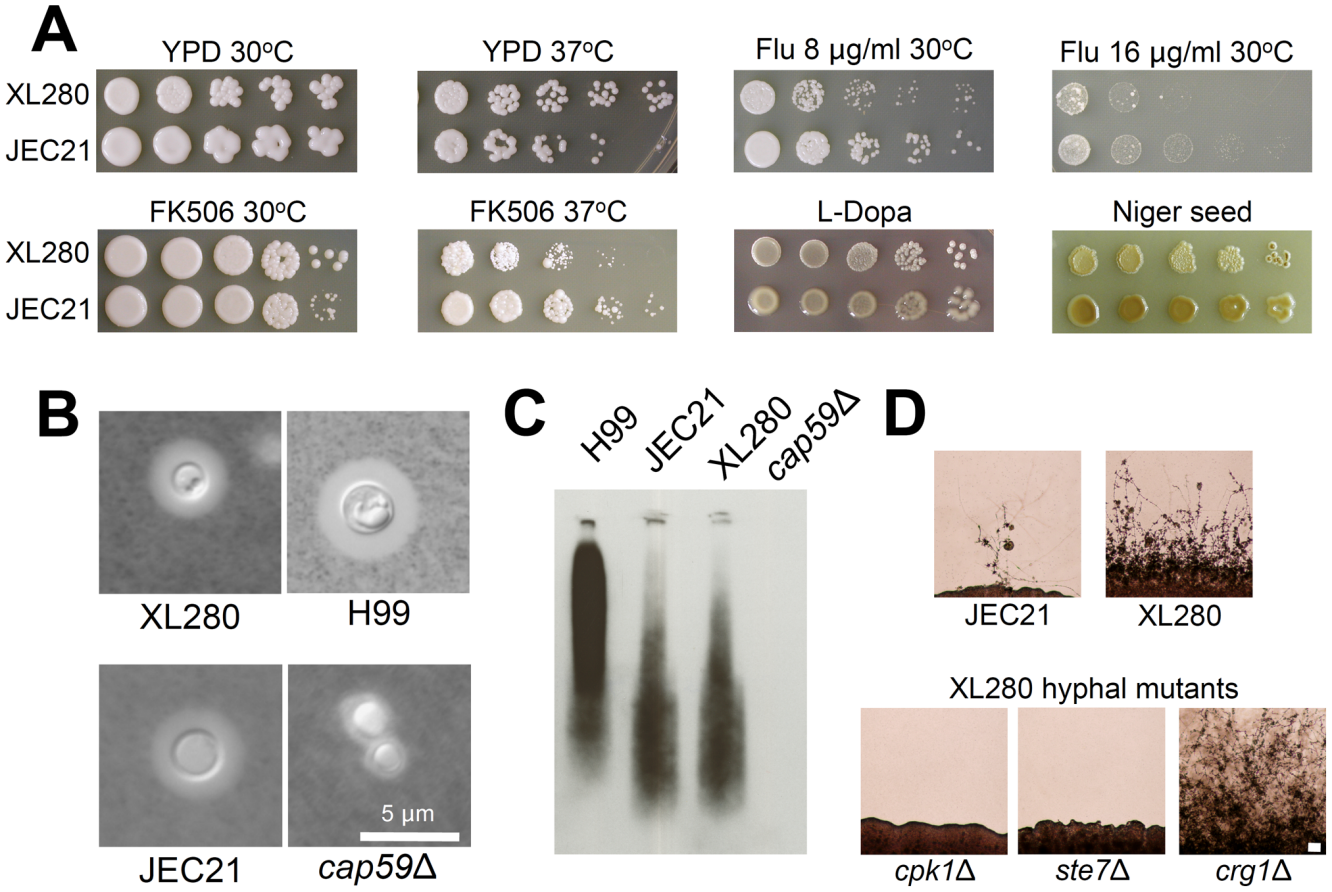
Phenotypes of virulence factors. In vitro phenotypic assays of virulence associated attributes of XL280α and JEC21α. (A) The strains were grown in liquid YPD, washed with water, and spotted in 10-fold serial dilutions on the following media and conditions: YPD at 30°C and 37°C for 2 days, YPD plus 8 or 16 µg/ml of fluconazole at 30°C for 6 days, YPD plus 1 µg/ml of FK506 at 30°C and 37°C for 3 days, L-Dopa at 30°C for 3 days, and niger seed at 30°C for 6 days. (B) For capsule evaluation, cells were grown in DMEM for 6 days and visualized by light microscopy using India ink and (C) supernatants of the cells containing secreted capsule were isolated, subjected to gel electrophoresis, and then immunoblotted with an anti-GXM antibody. (D) Unisexual reproduction cultures were incubated on V8 agar (pH = 7), for 2–3 weeks in the dark at room temperature. Scale bar = 100 µm.

*Cryptococcus* must evade and escape the immune system to successfully colonize the respiratory tract of the host and disseminate to the CNS. Thus we assessed major virulence attributes (growth at 37°C, melanin production on niger seed or L-DOPA media, and capsule production), antifungal drug sensitivity (fluconazole and FK506), and unisexual reproduction (Figure 1). The strains grew equally well at higher temperature but exhibited growth differences in the presence of fluconazole or FK506 (Figure 1A). Melanin is a dark cell wall-associated pigment that is negatively charged and protects *Cryptococcus* from macrophage killing and oxidative and possibly also nitrosative stress challenge [52], [53]. Melanin production was similar on L-DOPA (data not shown), whereas JEC21α exhibited higher levels of melanin on niger seed medium.

Capsule is a polysaccharide structure that can inhibit phagocytosis of *Cryptococcus* and also enhances intracellular replication of yeast cells that have been phagocytosed by macrophages [54], [55]. Capsule secretion and attachment to the cell wall are essential for virulence and acapsular mutants (i.e. *cap59*Δ) are severely attenuated/avirulent in disease progression models [43], [56]. XL280α and JEC21α were equally capable of secreting and attaching the capsule on their cell wall under capsule-inducing media and under physiological conditions (DMEM at 37°C under 5% CO_2_) (Figure 1B and 1C). However, we did observe a slower migration of secreted capsule in the serotype A strain H99 that may play a role in the pathogenesis of this strain [57]. The composition and structure of the capsule is different between serotypes and is usually used as a serotype classification tool of uncharacterized *Cryptococcus* strains. Even closely related strains may exhibit a different migration pattern of secreted capsule due to variations in capsule structure [58].

A major difference was observed in hyphal development during α-α unisexual reproduction (Figure 1D). As previously reported, JEC21α generates only a limited amount of hyphae after prolonged incubation (∼2 weeks) on mating media, manifested as only one or a few tufts of hyphae along the growth periphery [8], [59]. In contrast, XL280α is hyperfilamentous with abundant hyphae emerging as soon as 24 hrs after plating on mating media that emerge along the entire circumference of the growth periphery. The pheromone-signaling cascade regulates hyphal development during sexual reproduction. Several components have been found to play a major role in filamentation. Crg1 is an RGS protein and a negative regulator of the pheromone-signaling cascade and cAMP pathway; mutation of the gene enhances hyphal development and virulence [60]. In previous studies we isolated an insertional mutant of *CRG1* in the XL280α background, which is more hyperfilamentous than the WT during unisexual reproduction [47] (Figure 1D). On the other hand, Cpk1 and Ste7 are a highly conserved MAP kinase and MAP kinase kinase that play central roles in the pheromone response pathway [42], [46], [47]. Deletion of these genes causes a severe defect in hyphal development during unisexual reproduction (Figure 1D).

Thus, apart from hyphal development, the phenotypic analysis revealed that the major virulence factors of XL280α and JEC21α *in vitro* are similar and a possible impact on virulence may be attributable to the differences in hyphal development or other attributes not analyzed here.

### XL280α is a hypervirulent serotype D strain

**Figure 2:**
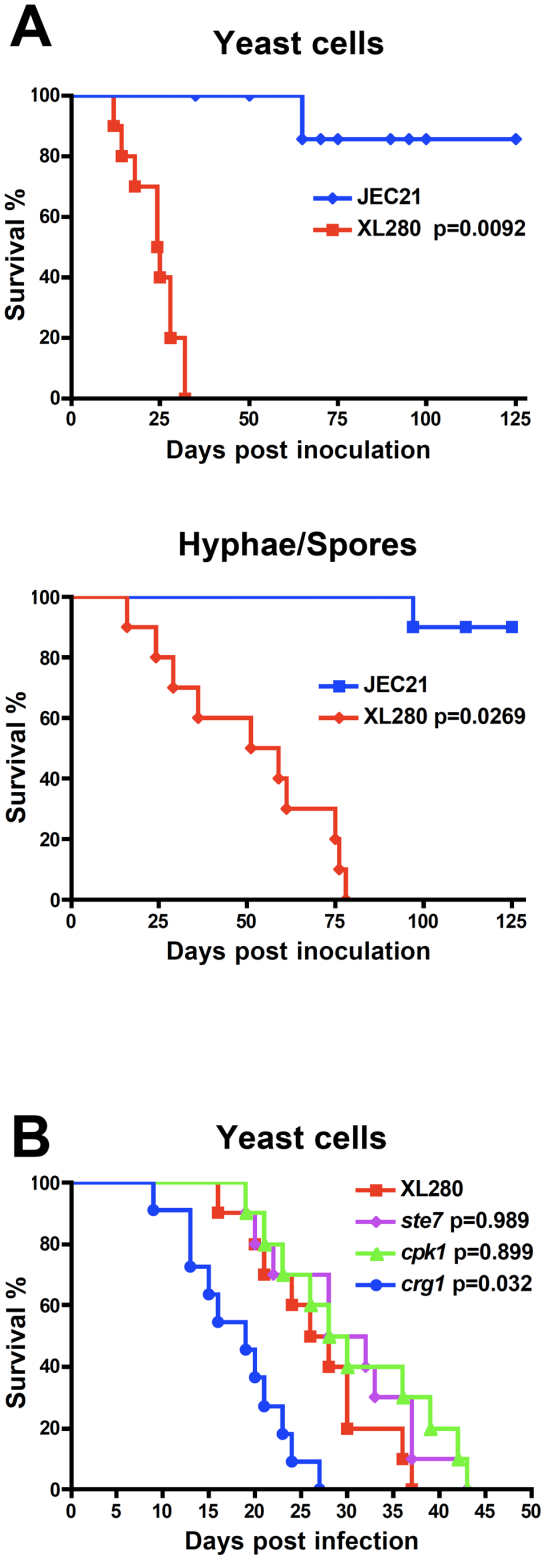
XL280α is hypervirulent compared to JEC21α. 10 DBA mice were infected intranasally with 1×10^6^ yeast cells or a mixture of yeast and spores isolated from a unisexual reproduction culture from each strain. Animal survival was monitored for 125 days post infection. (**A**) The XL280α strain was significantly more virulent than JEC21α in both cases (*p* = 0.0092 and *p* = 0.0269). (**B**) The components of the pheromone signaling cascade are dispensable for virulence in the XL280α background (*ste7 p* = 0.989 and *cpk1 p* = 0.899). However, the *crg1* mutant was significantly more virulent than WT (*p* = 0.032). Statistical analysis was performed using the Log-rank (Mantel-Cox) test.

Previous studies showed that even a low inocula of XL280α yeast cells (5×10^5^ cells/ml) displayed high levels of virulence in the murine model [39], [40]. Virulence assays were performed using the inhalation instillation infection model to compare the relative pathogenicity of XL280α with JEC21α. The mice were infected with one dose (1×10^6^ CFU/animal) using either yeast cells or a yeast/spore mixture produced by a unisexual reproduction mating culture incubated on V8 media to stimulate sexual reproduction. Survival was monitored for 125 days after infection. We found that XL280α was significantly more virulent than JEC21α (Figure 2). Mice infected with an XL280α yeast inoculum succumbed to the disease by 35 days post infection, while in the yeast/spore mixed infection the animals progressed to lethal infection by 80 days. The lower pathogenicity of the yeast/spore mixture may be attributable to the presence of hyphae generated during mating prior to infection, which have been associated with low virulence in previous studies [22], [40]. On the other hand JEC21α was severely attenuated for virulence in both cases. The first signs of pulmonary cryptococcosis (tachypnia, weight loss) were observed as early as 6 days post infection in mice infected with XL280α. We also noticed that moribund XL280α infected mice exhibited severe neurological symptoms often associated with cryptococcal meningitis (disorientation, balance disorder, and hydrocephalus due to accumulation of cerebrospinal fluid [CSF]; data not shown). However, these were not observed in any of the JEC21α infected animal cohorts. These findings indicate that XL280α is highly pathogenic in the murine model, compared to the hypo/avirulent strain JEC21α.

Previous studies have shown that there is a genetic link between virulence and the signaling pathways that regulate morphogenesis. It has been shown that virulence and hyphal development are quantitative traits and some components of the pheromone signaling cascade are involved in pathogenesis. However, the majority of hyphal development genes tested were found to be dispensable for virulence in the low virulence serotype D strains [22], [38], [42], [60], [61], [62], [63]. We therefore tested several well characterized genes known to be involved in hyphal development. Mice were infected intranasally with XL280α WT and *crg1*, *ste7*Δ, and *cpk1*Δ mutant strains. Virulence of the *ste7*Δ and *cpk1*Δ mutant strains was comparable to the WT, while the *crg1* insertion mutant was more virulent than the WT (Figure 2C) [60]. These phenotypes are consistent with previous observations in both the hypovirulent serotype D and the highly virulent serotype A strain backgrounds [42], [46], [64].

### Progression of XL280 infection

**Figure 3:**
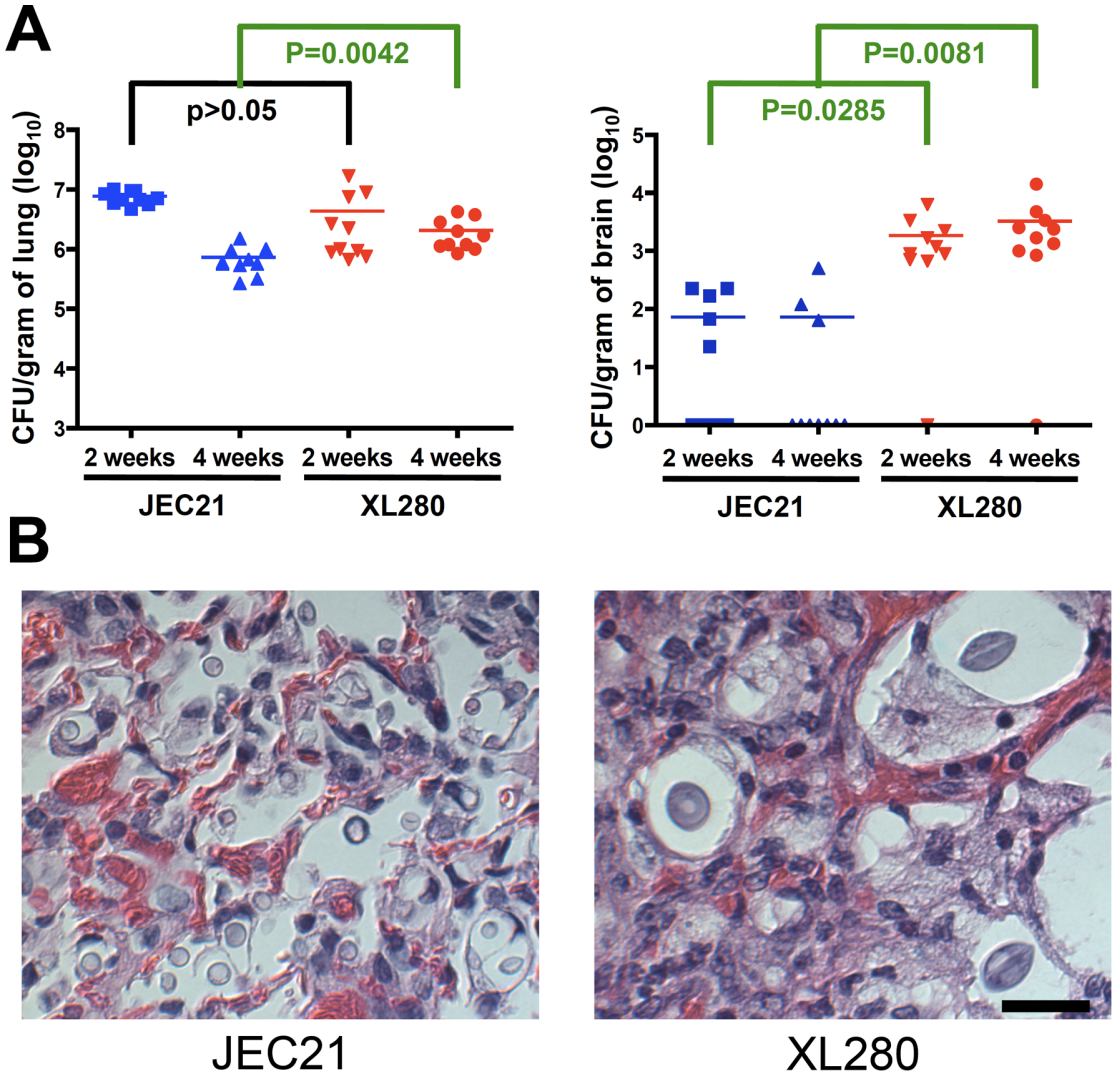
XL280α disseminates to the brain. (A) Mice were infected intranasally with 5×10^5^ yeast cells. At two and four weeks post infection the lungs and brains were isolated from 10 animals infected with each strain. The tissues were homogenized and serial dilutions were plated to recover CFUs and calculate the fungal tissue burden. XL280α fungal burden was significantly higher in both the lung and brain at four weeks post infection (*p* = 0.0042 and *p* = 0.0285). (B) Based on histopathological analysis of pulmonary tissues, XL280α yeast cells were morphologically different from JEC21α. They were significantly larger and exhibited an oval structure. Scale bar = 20 µm.

Although strain JEC21α is attenuated for virulence, during the course of the experiment a few mice exhibited signs of temporary distress that are typically associated with pulmonary infections. Thus, we hypothesized that the two strains might cause disease in different ways. To investigate the progression of disease during XL280α and JEC21α infections, growth and dissemination of the fungal cells to various organs were examined. Ten mice for each group were infected through intranasal instillation using a 2-fold lower inocula of yeast cells (5×10^5^ CFU/animal). This dose served to delay the pathogenicity of XL280α and allowed monitoring of the progression of the infection over the course of a month. Therefore, we measured the prevalence of the cells at an early time point in the infection course (2 weeks) and a later one (4 weeks). Higher numbers of CFUs in the lungs are indicative of a predominant pulmonary infection while presence of CFUs in the brain reflects dissemination to the central nervous system. We found that both strains were able to survive inside the host, and their levels in the lung were similar at two weeks, although by four weeks JEC21α was considerably less prevalent (Figure 3A). Interestingly, XL280α was at a significantly higher abundance in the brain at both time points, while JEC21 infection was cleared from the CNS by four weeks post infection (Figure 3A). These results indicate that XL280α and JEC21α are both capable of surviving and replicating inside the host; however, XL280α more successfully establishes an infection in the lung and then disseminates to the central nervous system to cause meningoencephalitis.

XL280α and JEC21α both grow as a yeast on solid and in liquid media under laboratory conditions. However, XL280α produces abundant hyphae during solo incubation on mating inducing media leading to unisexual reproduction. Although physiological conditions inhibit hyphal development inside the host, there are rare cases where hyphae have been observed to develop during infection [22], [25], [26], [27]. To examine the fungal morphology of XL280α and JEC21α, mice were infected intranasally, and histological examination of the lungs and brains was conducted at two and four weeks post infection. As expected hyphae or pseudohyphae were absent from the samples and only yeast cells were observed with both strains (Figure 3B). However, in the lung tissues of *Cryptococcus* infected mice the morphology of the XL280α cells differed from the typically round yeast cells observed *in vitro*. The cells were more elongated, oblong, and resembled oval like structures (Figure 3B). We excluded the possibility that this cell morphology was an artifact caused by the fixation or staining process, as tissues from both XL280α and JEC21α infected mice were processed by the same technical procedure. Moreover, this phenotype was observed in multiple independent experiments, different animals, and at different time points. However, we were not able to observe this morphology under laboratory conditions outside the host. These oval shaped cells seemed larger than their counterpart round yeast cells, which could contribute to the virulence of this strain. Previous studies have shown that morphological changes that result in giant/titan cells affect the virulence of *Cryptococcus* and increase pathogenicity [20], [21], [65].

### XL280 infected animals exhibit a Th2 biased cytokine profile

Previous studies have shown that the cytokine profile of *Cryptococcus* infection differs significantly between the different lineages and is usually indicative of the immune response and the outcome of the disease. A recent study showed that cytokine expression during *C. gattii* infection in mice induces a Th2-type immune response and it has been hypothesized that *C. gattii* lung infections are not efficiently cleared by the immune system of immunocompetent individuals because they fail to induce a protective Th1-type immunity [32], [66]. On the other hand, previous studies concluded that *C. neoformans* var. *grubii* isolates elicit both Th1 and Th2 immune responses in healthy individuals, which are resistant to serotype A infections. The balance of the associated cytokines triggers a synergistic effect between Th1 and Th2 immunity that clears the infection and promotes the development of a protective immune response [67], [68], [69]. These findings are in accord with *C. gattii* infections occurring commonly in immunocompetent hosts, while *C. neoformans* is most frequently associated with infections in immunocompromised individuals.

**Figure 4:**
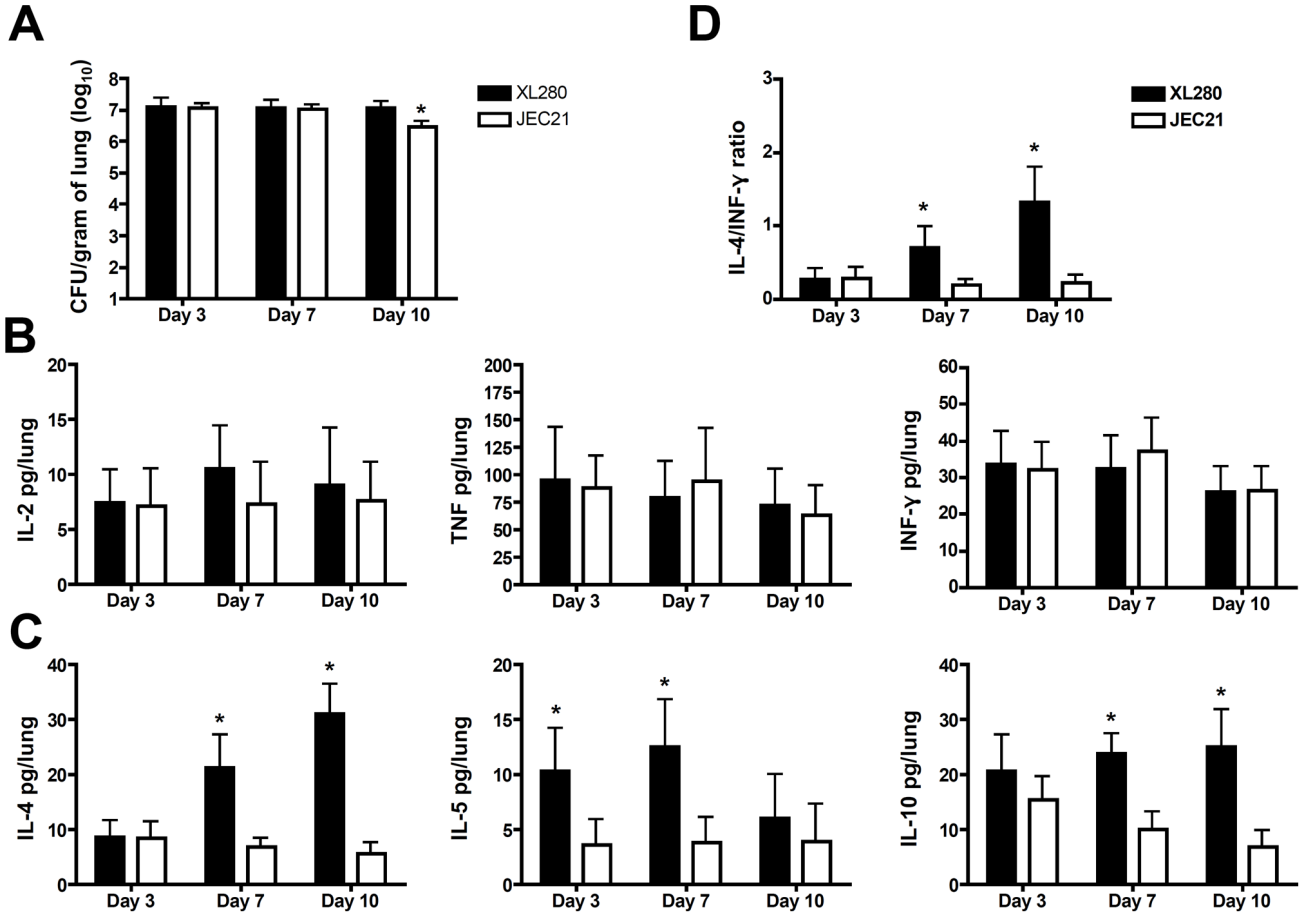
Immune response to XL280α is polarized towards Th2-type immunity. Mice were infected intranasally with 1×10^6^ yeast cells and cytokine production was assayed at 3, 7, and 10 days post infection. (**A**) The lungs were isolated, homogenized, serially diluted, and plated for CFUs. (**B**) The Th1 cytokines IL-2, TNF, and IFN-γ were similar between XL280α and JEC21α infected mice. (**C**) In contrast the Th2-type associated cytokines IL-4, IL-5, and IL-10 were significantly higher in XL280α infected mice. (**D**) The ratio of IL-4/IFN-γ was significantly higher in XL280α infected animals at 7 and 10 days post infection. The data represents four independent experiments. The asterisk (*) signifies a *p*<0.05, derived from two-way ANOVA statistical analysis.

To determine the cytokine profile during infection by XL280α or JEC21α, mice were infected with yeast cells from each strain (1×10^6^ CFU/animal) and lungs were collected at three time points early in the course of infection (3, 7, and 10 days post infection) (Figure 4A). No significant differences were apparent for the majority of cytokines expressed in XL280α and JEC21α infected mice (Figure 4B). However, IL-4, IL-5, and IL-10 levels were significantly higher (p<0.05) in XL280α infected mice compared with JEC21α (Figure 4C). All three cytokines have been associated with Th2 immunity; IL-4 is a major determinant of the differentiation of T cells into Th2 cells, while IL-10 can downregulate Th1 responses. The balance between Th1 and Th2 cytokines defines the progression and the outcome of the disease. To assess the overall Th1/Th2 cytokine balance the ratio of the IL-4 Th2 cytokine to IFN-γ, a Th1 associated cytokine critical for innate and adaptive immunity, was calculated. At 7 and 10 days post infection cytokine production was significantly polarized towards Th2 immunity in XL280α infected mice (Figure 4D). These findings are in accord with our observation that XL280α, a serotype D strain associated with high mortality, is able to evade the immune system and successfully disseminate to the CNS. Thus, we hypothesize that XL280α induces an immune response that is polarized towards Th2 immunity and higher levels of IL-4 may downregulate cell-mediated immunity and host protection and as a consequence exacerbate the course of disease progression.

### Histological analysis of XL280α and JEC21α pulmonary infections

**Figure 5:**
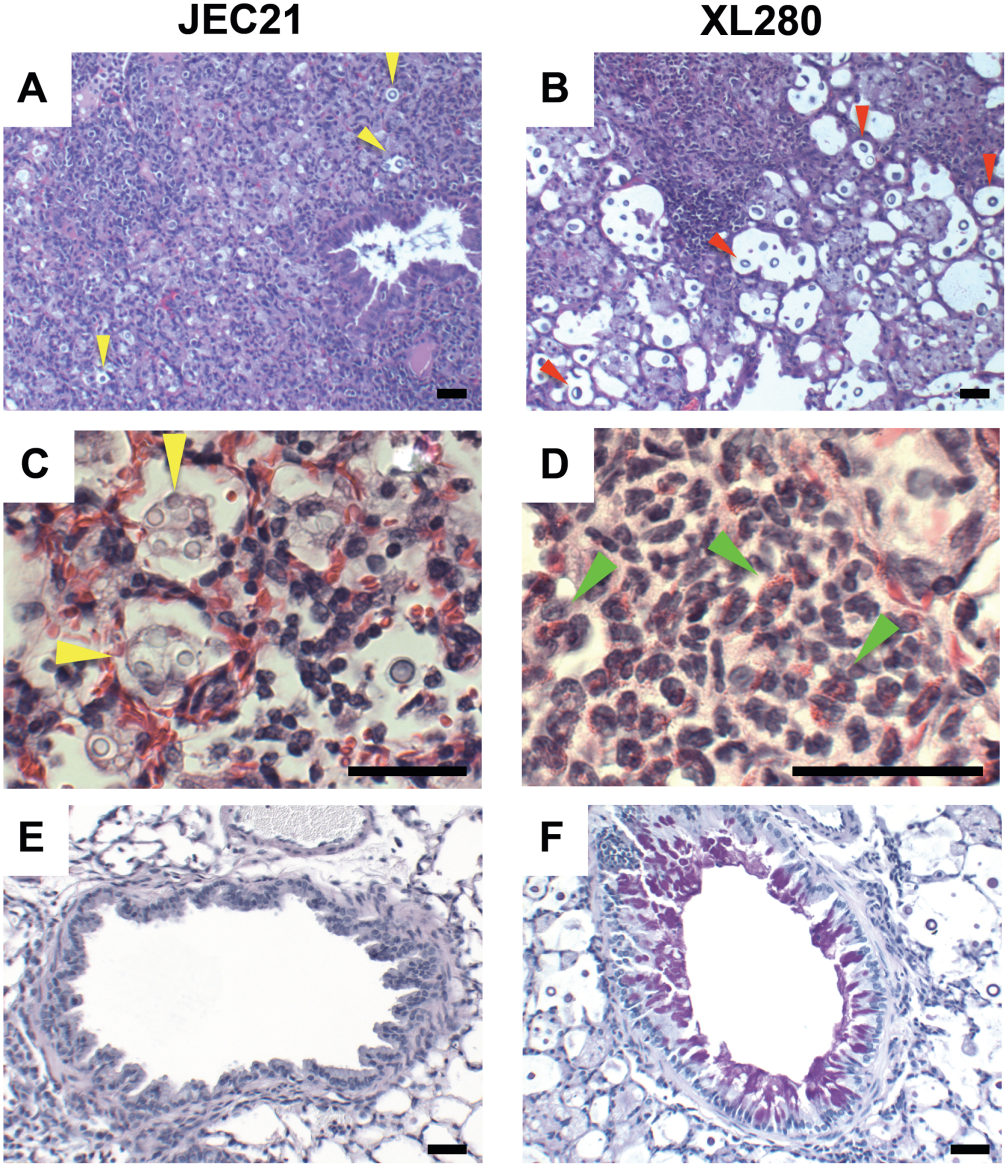
XL280α infection results in eosinophil infiltration and goblet cell metaplasia. Lungs of mice challenged with JEC21α and XL280α were isolated 10 days post infection, fixed in formalin, and processed for histological analysis. Tissue sections were either stained with Hematoxylin and eosin (H&E) stain and photographed with a 10× (A and B), 40× (C and E), or 60× objective (D), or stained with Periodic acid–Schiff (PAS) stain and photographed at 20× (F). JEC21 caused marked inflammation in the lung (**A**) with widespread growth of *Cryptococcus* and enlarged macrophages with proliferating intracellular cryptococci (yellow arrows) (**C**). Airways and vessels exhibit normal goblet cell growth (PAS-negative) (E). In contrast, XL280α yeast cells (red arrows) stimulated a diffuse inflammatory response (**B**) with pulmonary eosinophilia (green arrows) (**D**) and PAS-positive goblet cells (**F**). Internalized yeast cells were not detected in XL280α infected pulmonary tissues. Scale bar = 50 µm.

Our cytokine analysis revealed a Th2 biased production during XL280α infection. To determine whether XL280α challenged mice developed lung lesions indicative of an allergic Th2 bronchopulmonary response, we isolated lung tissues from JEC21α and XL280α infected animals 10 days post infection. Histological sections were stained with hematoxylin/eosin and mucicarmine and analyzed by light microscopy. The overall pathology was significantly different between the two strains. In the lungs of mice infected with JEC21α the cellular infiltrate surrounding the airways appeared to be densely packed with leukocytes, mostly neutrophils and macrophages, forming tight inflammatory foci (Figure 5A). Although cryptococcal growth was observed to be widespread, it appeared to be contained in these inflammatory foci of the infected lung. Interestingly, a majority of the JEC21α cells appeared to be within macrophages with some of them harboring more than one *Cryptococcus* cell (Figure 5C, yellow arrows). In contrast, the cellular infiltrate appeared more diffuse in the lungs of XL280α infected animals, with scattered dense areas of leukocytes characterized by the presence of numerous eosinophils (Figure 5D). Growth of XL280α cells was widespread around the bronchiolar and alveolar airspaces, and observed both within and outside of inflammatory foci (Figure 5B). Surprisingly, XL280α cells appeared to be resistant to phagocytosis, as we were not able to observe any macrophages containing yeast cells (although macrophages were widespread in the lung tissues). In addition, we observed changes in the airway morphology of the lung. XL280α infected mice displayed goblet cell metaplasia surrounding small blood vessels and airways, stimulating extensive mucin secretion, which was absent in JEC21α infected lungs (confirmed by PAS staining) (Figure 5E and Figure 5F). Diminished leukocyte infiltration, goblet cell metaplasia, and recruitment of eosinophils at the site of infection are all indicative of a non-protective Th2 immune response to XL280α but not JEC21α.

Based on cytokine analysis and lung pathology we conclude that XL280α induces an immune response consistent with less protective Th2 polarized immunity, which potentially exacerbates cryptococcal infection. Moreover, the significantly larger size and the unique morphology of XL280α cells may impair phagocytosis by macrophages, and thereby allow the cells to grow and proliferate inside the host and successfully disseminate to the brain.

## Discussion

*Cryptococcus neoformans* causes lethal infections of the central nervous system in immunocompromised individuals. The A and D serotypes contribute differentially to the magnitude of infection by this organism. The vast majority of these infections worldwide (∼95%) are caused by highly virulent strains of serotype A [50], [70]. A direct comparison of two widely used serotype A and D laboratory strains, H99α and JEC21α, showed that H99α responds differently to stress, produces more melanin, exhibits higher rates of proliferation inside the host, and is significantly more virulent than JEC21α [10], [50], [71]. In healthy individuals serotype A and D isolates induce a strong inflammatory immune response that clears the infection and prevents the fungus from disseminating to the brain and causing meningoencephalitis. In this study we report a serotype D strain, XL280α, which is significantly more virulent than its parental strain JEC21α. Moreover, we found that the pathogenicity of XL280α is comparable to the highly virulent *C. neoformans* strain H99α based on previous studies. Surprisingly, XL280α induces a less protective immune response during pulmonary infection, which is characterized by high levels of Th2 type associated cytokines in the lung. Furthermore, lung pathology confirmed a polarization towards Th2 immune response based on the presence of eosinophils and goblet cell metaplasia in XL280α infected mice.

Serotype D clinical isolates are less common compared with the highly virulent serotype A clinical strains that usually infect immunocompromised individuals. Moreover, previous studies showed that serotype D strains (including JEC21) often exhibit lower virulence *in vivo* and usually require higher inocula for intranasal instillation or tail vain injection in a murine infection model [44]. However, in previous studies Zhai *et al*. and we showed that even low relatively low inocula of XL280α could successfully infect and cause disease in mice [39], [40], similar to what we have observed for highly virulent serotype A strains [72].

XL280α is a serotype D strain derived from the cross of two sibling strains B3501α and B3502**a** [38]; B3501α/B3502**a** are F1 progeny that descended from a cross between a clinical (NIH12α) and an environmental isolate (NIH433**a**) [44]. In recent studies, the genomes of XL280 and strain JEC21 (JEC21α/JEC20**a** are congenic with the XL280α parental strain B3502**a**) were compared by next-generation sequencing (NGS) revealing that the two strains are genetically identical across 81% of the genome, the remaining 19% differs by 0.5%, and overall they are 99.88% identical at the sequence level [39]. Therefore, it was expected that the two strains would behave similarly in a variety of phenotypic assays, including virulence associated phenotypes. However, a major difference is that XL280α is hyperfilamentous compared to JEC21α. During unisexual reproduction, XL280α generates abundant hyphae around the circumference of the colony, while JEC21α hyphae are scarce and limited. Extensive genetic, molecular, and genomic analysis showed that XL280α unisexual hyphal development is characterized by a mixture of haploid or diploid hyphae that generate abundant haploid recombinant spores that are the infectious propagules of *Cryptococcus* [18], [39], [47]. Interestingly, it has been proposed that the unique ability of α strains to generate hyphae and spores during unisexual reproduction may contribute to the predominance of this mating type in clinical isolates [11]. However, unisexual reproduction has been directly observed under laboratory conditions only for the less pathogenic strains of serotype D. Surprisingly, we found that the serotype D hyperfilamentous XL280α strain is hypervirulent in the murine model compared with JEC21α. The pathogenicity of XL280α has been previously reported; however, there was no direct comparison to other serotype D strains [39], [40]. In this study we show that XL280α is highly pathogenic and the spores produced during unisexual reproduction can spread, germinate, and potentially infect and kill a host.

Hyphal development in *C. albicans* is essential to survive inside the host; nonfilamentous mutants are attenuated for virulence in the murine model [73]. During infection *C. albicans* hyphae invade epithelial and endothelial cells causing severe tissue damage and gaining access to the bloodstream resulting in dissemination of the fungus to various organs [74], [75], [76]. More importantly, yeast cells engulfed by macrophages switch to hyphae that perforate the mammalian cells and enable the fungus to escape and survive inside the host [77]. Although *C. neoformans* hyphae are rare *in vivo* and are associated with low virulence, pseudohyphae have been observed in histological samples and they were found to confer resistance to *Acanthamoeba* spp. phagocytosis [27], [78], [79], [80]. Although pseudohyphae are dispensable for virulence [81], stochastic transitions from yeast to pseudohyphae may serve as an escape strategy and confer resistance to phagocytosis by mammalian macrophages similar to giant/titan cells.

Interestingly, the progression of the disease was quite different between the two strains. Fungal proliferation was observed in the lung and the brain for both XL280α and JEC21α early in the infection (two weeks); however, the fungal burden for XL280α was significantly higher in both organs at a later time point (4 weeks). In addition, moribund mice infected with XL280α exhibited severe neurological defects associated with central nervous system disorders. This type of phenotype is often associated with *C. neoformans* infections that primarily cause severe cryptococcal meningitis, while pulmonary infections are more common in *C. gattii* infected individuals [82], [83]. A recent study found that *C. neoformans* preferentially disseminated to the brain during infection, while *C. gattii*, which fostered a fatal lung infection, failed to cause lethal meningoencephalitis in two animal models [84]. These results confirm clinical observations where 70% of *C. gattii* cases suffer from pulmonary infections and only 7.8% of patients presented with CNS infections [83]. *C. neoformans* is known to elicit marked inflammation in the lung, which could inhibit proliferation in pulmonary tissues and facilitate migration to the CNS. On the other hand, the immunosuppressive nature of *C. gattii* may allow it to evade the immune system and preferentially establish an infection in the lung. Our results suggest that the serotype D strain XL280α fosters an infection in both organs that possibly contributes to its pathogenicity.

We also found that XL280α cells were significantly larger than JEC21α inside the host, and adopted an oval morphology that was widely observed in multiple independent samples and at different time points. Based on lung pathology, we also observed that XL280α cells were resistant to phagocytosis by macrophages. Previous studies have shown that giant/titan cells observed during cryptococcal infections are resistant to phagocytosis and can contribute to pathogenesis [21], [65]. The heightened virulence of the XL280α strain might be attributed to higher levels of capsule produced by these larger cells, which may negatively impact the immune system of the host. Moreover, this unique morphology and the larger size of the cells may confer protection against phagocytosis by alveolar macrophages and allow more rapid dissemination to the CNS. Although capsule production was similar between JEC21α and XL280α *in vitro*, the larger size of XL280 *in vivo* may result in significantly higher levels of capsule volume, which is known to protect against oxidative stress and may have contributed to the survival of the strain. Previously, it has been reported that rapid changes in cell size and capsule structure, occurring in pulmonary tissues, are associated with tissue invasion and may favor crossing of the blood-brain barrier [85], [86]. These alterations in cell surface composition may enhance organ invasion and cause higher affinity of *Cryptococcus* cells to the brain.

Our findings suggest a different pattern of infection between XL280α and JEC21α. The immunological response elicited by pathogens can greatly affect the outcome of the disease. Host defense against *Cryptococcus* is mediated by T cell mediated immunity. Previous studies have shown that the different species of *Cryptococcus* induce distinct immune responses inside the host [28], [31], [32], [67]. In this study we found that the cytokine profile of XL280α infected mice is shifted towards Th2 type immunity. The major Th2 associated cytokines, IL-4 and IL-5, were elevated in lungs of mice infected with XL280α and the ratio of IL-4/TNF-α was significantly higher than for JEC21α. In addition, histopathological analysis confirmed the presence of numerous eosinophils and goblet cell metaplasia around the airways in XL280α infected lungs, indicative of Th2 immunity. Interestingly, bronchovascular infiltration was diffuse reflecting less inflammation in mice infected with XL280α. On the other hand, JEC21α caused a strong inflammatory response, with massive infiltration of neutrophils and macrophages. At day 10 post infection the majority of JEC21 cells were within macrophages in these inflammatory foci. These findings suggest that XL280 thrives inside the host, possibly because it fails to induce protective inflammation during infection. Surprisingly, this phenotype is similar to *C. gattii* infections that skew the immune response towards Th2 immunity, which allows them to evade the immune system, proliferate in pulmonary tissues and establish a fatal infection, if untreated, in healthy individuals [32]. We therefore consider the possibility that XL280α similarly evades the immune system, by inducing a Th2 polarized immune response, and resisting phagocytosis by macrophages. These conditions allow the cells to survive and proliferate in the lung before disseminating to the brain. It is also possible that XL280α failed to provoke migration of neutrophils to the lung, which are essential for protective immunity and were observed in JEC21α. We note that our observations are different from those usually reported for *C. neoformans* infections. The virulent strains of serotype A are known to induce Th1 and Th2 protective immunity in healthy individuals, while serotype D induced immune response is not considered of high medical importance due to the lower pathogenicity of this group [28], [29], [30], [32], [87], [88]. However here we report a serotype D strain that is highly virulent and able to skew the immune response towards a less protective Th2 immunity inside the host. XL280α is an F2 progeny derived from NIH12α (clinical isolate) and NIH433**a** (environmental isolate). The differences we observe in this hyperfilamentous strain in terms of morphogenesis and virulence are due to meiotic recombination between the genomes and possibly also novel genetic diversity introduced during bisexual reproduction (as has been previously reported [39]).

Taken together, our results provide novel insights into pathogenicity of *C. neoformans*. This the first report of a hypervirulent strain of the serotype D lineage that induces less protective immunity during infection, similar to *C. gattii* in otherwise healthy individuals. Our findings suggest that other clinical serotype D isolates may share similar virulence attributes that may underlie and explain their more frequent occurrence in certain geographic regions, such as Europe.
